# A Glance at Medical Science among Some of the East Indian Nations

**Published:** 1860-04

**Authors:** 


*A Glance at Medical Science among some of the East Indian Nations.* From the French of Dr. L. S. HEYMANN.

The prescriptions of the Javanese doctors, called *dukum* in the Malay tongue, are always very complicated, consisting of ten, twenty, or a larger number of drugs, each one destined to combat one of the most prominent symptoms of the disease under treatment. Among the substances which make up the Materia Medica of the Javanese, there are some possessed of incontestable pharmaceutical virtues. Some of these have been recently tested in the hospitals of Java, and in similar establishments on the other isles of the Sound, and the experiments have been successful.

The Javanese physicians do not trouble themselves about the diet and regimen of their patients; but, on the other hand, they administer drugs as frequently to those who are well, to preserve their health and prevent disease, as with the view of curing it. Chiefs and persons of high station are incessantly occupied with the condition of their health, and consequently take daily different medical compounds. Among others, there is an officinal preparation, called *Djamoe*, which is employed largely by those who desire to protect themselves from all kinds of disease, and which is composed of from fourteen to twenty-six different substances. It is not employed until the age of fourteen years is attained, but other compounds, more or less analogous, are used for children. The child in the cradle is exposed to the necessity of using these, while the most decrepit old men also demand of *djamoe* additional years of life and health. The following articles enter into the composition of Djamoe: Carum carui, Meeso-i, Cinnamomum sintoc, Sprantu, Semen Coriandri, Nux moschata, Anethum graveolens, Alumen, Capsicum bicolor, Piper cubeba, Piper nigrum, Kedawung, Caryophillus aromaticus, Citrus limonellus, Asafœtida, Rad. Glycyrrhizæ, Cort. Cinnamomi, Kajuang-ngien, Kæmpferia rotunda, K. galanga, Allium cepa, Ocymum basilicum, Drymariæ, Klabet, Flores Bixæ Orellanæ, and Djongrahab.

*Djamoe* cannot be an inert preparation. The different articles are mixed in definite proportions, always represented by odd numbers, to which great cabalistic importance is attached. They are pulverized on a smooth stone, moistened with lemon-juice and holy water; verses of the Koran are recited during the manipulation, and finally boluses are formed, which are administered in the morning. A more simple djamoe contains only eleven of the substances already mentioned, with the addition of Calamus aromaticus, Acorus calamus, and *temu-lawak.* Aside from its preservative qualities, djamoe is said to be very efficacious in intermittent fever and colic. If the fever does not yield to three or four boluses administered the first day, the lemon-juice, employed in the preparation of djamoe, is replaced by urine. In the treatment of colics, a little brandy is added, especially in frequently-recurring cases, where they are brought on by the use of green fruit.

Djamoe, prepared according to the first formula, is extensively used to produce abortion. This is not considered as an immoral or reprehensible act. Married women often procure abortion with the consent of their husbands, and without any effort to conceal it. It is difficult to say whether djamoe will alone produce abortion, as other means are employed along with it. However, when abortion is required, or the cure of disease, the consummate experience of some matron often renders the intervention of a *dukon* useless. There is no Javanese family which does not possess a supply of the most common medicines, contained in a casket, along with a stone plate and a kind of pestle—the only instruments employed in the preparation of medicaments. Scales and weights are not used—an approximative guess determines the dose.

Among the different mixtures which these domestic pharmacies contain is a panacea, frequently used, made of Kæmpferia galanga, K. rotunda, Curcuma rotund. et long., Allium sativum, and common salt.

These substances are triturated, and boluses are made, as in the case of Djamoe, which are administered at intervals of one or several hours. Frequently it is proposed, in this way, to produce abundant diaphoresis. It, however, produces, at times, colics and diarrhœa, and, if persevered in, dysenteric symptoms, which it would be considered very bad to treat immediately.

Such is the Therapeutics of the Javanese. As they rarely employ a medicament without associating it with a number of others, it is not possible to determine the special action of the different drugs. There are some, however, whose efficacy in certain affections has been demonstrated by the most exact experiments. Thus, the root of the Trebah Japan (*Rhinacanthus communis*) seems very efficacious in the treatment of Herpes circinnatus and H. phlyctenoides. A mixture is prepared, by rubbing it with vinegar, which is applied three times a day on the affected portions of the skin. It is ordinarily only necessary to repeat these applications twenty-one times to insure a cure. Among the drugs produced on the island of Java are some that have been long known in our own shops, viz.: cassia, croton, ricinus, &c. Although the Javanese principally seek their remedies in the vegetable kingdom, yet they employ a number of products obtained from the animal and mineral kingdoms—among the latter, copper and arsenic being frequently used.

The excrements of a large number of animals also enjoy great pharmaceutical repute; but the human urine is the most celebrated of all these. When an accouchement progresses slowly, the urine of the husband or of some one else is administered to the woman. This disgusting agent is also popular in the treatment of asthma, and, as a topical agent, it is considered the best of all collyria.

Dysentery has furnished an opportunity to the Javanese doctors to invent interminable formulæ. The treatment is always, and invariably, the same, and comprises a certain number of preparations which are to be successively administered. The compound first employed does not differ from djamoe; at the end of twenty-four hours, if there should be no amelioration of the symptoms, a decoction is made of a large number of substances, among which may be found rice and a large number of aromatic fruits, Cubebs and Capsicum longum. In case the patient is a child from five to ten years old, this decoction is substituted by a powder, which contains *album græcum* of the dog, that has been roasted at a fire and sprinkled with lemon-juice.

It is only when the resources of the domestic pharmacy have been exhausted that the patient is intrusted to the care of some medical notability of either sex. When the Javanese doctor approaches the patient, he fixes a look of attention or inspiration upon him; then pompously draws forth a bag containing the Djimat, (the formula of exorcism,) which is a band of paper covered with Arabic formulæ, and folds it with special care. This bag being placed upon the most painful portion of the patient's abdomen, he prepares the *Dukon*, (reciting, at the same time, a long series of prayers,) and places it under the ear of the patient, with one or more notes covered with hieroglyphs. This being done, he throws a nail, made red hot in the fire, or a magic stone, (*mustika*,) into the consecrated water, which the patent immediately drinks. For a last resort, when all other manœuvres fail, and the pitiless administration of a certain quantity of urine proves inefficacious, another *dukon* is employed. In some extremely rare cases, the treatment is finally intrusted to a European physician, who arrives almost invariably too late.

Blenorrhagia is somewhat common among the inhabitants of Java, and they have a large number of formulæ to combat it. In old cases, they employ frequently a mixture of cubebs, ginger, Alpinia galanga, coriander, white pepper, and nutmegs.

From the foregoing, it will be seen that the use of medicaments on all occasions is, among the Javanese, pushed to an extreme rarely met with elsewhere; but after an accouchement, the loosest reins are given to this mania. Whether the labor has been fortunate or not, the woman is covered with layers of countless drugs, either to favor the flow of the lochia, or to keep off all varieties of complication. The delivery is but accomplished, when incontinently a tisan is administered to the mother, prepared of wood cinders and tamarind pulp, or the midwife substitutes for this draught decoctions more or less complicated. The abdomen of the patient is covered with a host of ointments, whose composition varies according to the part for which they are specially designed; and this treatment is continued for weeks, although the most vigorous health of the mother would justify their discontinuance. The new-born child is also subjected to interminable manipulations. The skin is covered with a layer, several lines in thickness, of oils, balsams, and juices of plants—hardly any portion of the body escapes these applications.

Thus far we have written of the indigenous doctors. Java possesses, also, a number of Chinese doctors, and Chinese pharmaceutists. The shops of the latter compare favorably with those of the indigenous pharmaceutists, so far as order and neatness are concerned. Many of them are arranged like European pharmacies, and contain analogous preparations—the most of which are prepared of substances obtained from the vegetable kingdom The mineral substances employed by Chinese doctors are very few. They use, however, a number of substances obtained from the animal kingdom, to which marvelous virtues are attributed, and which are selected from the most disgusting products. For example: toad-flesh cures diarrhœa; that of the *Gecko*, tuberculous affections; that of the bat insures long life to him who uses it; while bat's blood and bile are reputed to cure syphilis, and its excrements are employed as an excipient in the preparation of certain pillular masses.

The skeleton of the scorpion, dried and pulverized, possesses diaphoretic virtues, and cures both rheumatism and small-pox. Horse-brain makes the hair grow; the heart of the same animal, dried and pulverized, strengthens the memory; his bones, pulverized, prevent sleeplessness; and the placenta of the mare cures amenorrhœa: but, in order that all these effects should be had, a *white* horse should be employed. Marrow from the bones of the ass, introduced, during sleep, into the ear, cures deafness; the horn of the rhinoceros arrests somnambulism, if placed under the ear while the somnambulist is asleep; pulverized and cooked in wine with the livers of the goose and duck, the same horn will cure hæmatemesis. Tapir-skin is used as a cushion and for mattresses by those who have vicious humors; and its urine is an antidote for copper poisoning.

In addition to the medicaments of known composition, the Chinese doctors of Batavia employ a large number of secret remedies brought from China, prepared with much elegance, and accompanied with attractive advertisements. These are employed to cure divers diseases, or to give convalescents that *embonpoint* which the Chinese value so highly; some have great reputation as aphrodisiacs, and are often employed to prevent impotence.

Most of the Chinese doctors in Java are not of pure extraction, and have never visited China, and hence they are less educated than their *confrères* of the Celestial Empire. But few escape the influence of the Javanese modes of practice. Equally superstitious, they play the part both of sorcerer and physician, and employ the agency of the stars or of a demon in their practice.

This is all very different in Japan. Medicine has there been cultivated with as much zeal as intelligence, and has attained a remarkable development. The old medical school, proceeding from the same stock as the Chinese school, and partaking of most of its doctrines, has not been slow in modifying these by contact, although limited in character, with Europeans, especially Hollanders and Portuguese. In spite of systematic governmental opposition to all innovation, many of the physicians appropriate freely the medical knowledge and doctrines of the foreigners with whom they come in contact; the study of the European languages opens up to them our scientific works, which they seize hold of with the sole desire of gaining information, which is one of the characteristic traits of the nation.

When the new school, during the second half of the sixteenth century, contended advantageously against the old traditions and European Pharmacology, it would have succeeded entirely, had it not been for the changes which followed the invasion of the Portuguese. The movement suffered thus a period of arrest, which still exists. Nevertheless, the government suffered the importation of a certain number of medicaments brought from Europe, which were obtained almost entirely from the vegetable kingdom. The number of these substances is very carefully limited, and every argument to increase them encounters insurmountable obstacles from the government. In exceptional cases, authority has been granted to import a medicine whose importation has not ordinarily been allowed, when the demand was made by a physician of renown.

China furnishes Japan a much larger number of medicines than Europe; from her all the rhubarb and opium are procured. There are, however, substances of analogous virtues to many of these medicines among the products of Japan. It possesses no vegetable that can take the place of the cinchonas; and quinine is hence allowed to be imported.

Among the inorganic substances, the Japanese doctors prepare some in accordance with the directions of European pharmacopœias: calomel, corrosive sublimate, sulphate and carbonate of magnesia, tartar-emetic, and the different salts of soda and potassa.

Most of the medicines used in Japan belong to the vegetable kingdom, and many of these are used for the same therapeutic indications as in Europe. Benzoin is employed as an expectorant and antispasmodic, opium and nux vomica as narcotics, catechu as an astringent, gamboge as a drastic, asafœtida as an antispasmodic and anthelmintic, myrrh as an excitant, stomachic, resolvent, and antiseptic; mastic is used in hæmoptysis, asthma, diarrhœa, and dysentery; sanguis draconis in hæmorrhages, dysentery, and chronic diarrhœa.

The mineral substances most frequently employed are as follows: Cinnabar, employed especially in syphilis. It is given in dose from 50 to 75 centigrammes, in cigarettes. Three or four such cigarettes are smoked daily, in order to bring on profuse salivation. Mercury rubbed up with syrup, so as to destroy the globules, is also extensively employed, in the form of pills, in small-pox; and also a mixture of mercury and alum, known as *kei-hun.* Tin filings, mixed with syrup, are used as an anthelmintic; and sulphate of iron, dissolved in water, as a hæmostatic.

A number of substances obtained from the animal kingdom are also to be found in the Japanese Medical Arsenal. Musk serves to combat spasms. Certain serpents, carbonized on a slow fire and then pulverized, are used in the treatment of syphilis, lepra, and chronic exanthems; an analogous preparation, made with *Gecko*, is employed in affections of the kidneys and bladder. Decoction of toads, frogs, and salamanders is reputed to cure convulsions, epilepsy, intestinal worms, chronic catarrhs, croup, tubercles, dropsy, and constipation; powders are likewise prepared from these animals, which are applied on tumors, ulcers, and different cutaneous lesions. The fat of the toad, called *Fiki-Kaheruabura*, is reputed to be very effective in preventing accidents during dentition.

The *cantharis* of Japan is smaller than the species used in Europe, but more active. This is used in vesecation, and is administered, in form of powder or infusion, in divers chronic affections of the genito-urinary organs. The Japan leeches are also very small, but they are rarely used.

The indigenous medical drugs are divided, by the doctors of the new school, into five classes, in accordance with their therapeutic action, viz.: acrid, tonic, and astringent; excitant and roborant; nervine and narcotic; resolvent and nutrient. These are distinguished by the Japanese or Chinese names, although most of the physicians are familiar with the names employed in European Pharmacopœias. They administer the medicines as decoctions, infusions, powders, and pills; and tinctures, electuaries, and extracts are also employed. There are no regular Pharmacies in Japan. Drugs are sold in their natural condition, either wholesale or retail, in the shops, where any one, whether professional or layman, can purchase. The physicians prepare and dispose of the various preparations which they prescribe. —*Gazette Hebdomadaire.* L. H. S.

